# *Geminal* parahydrogen-induced polarization: accumulating long-lived singlet order on methylene proton pairs

**DOI:** 10.5194/mr-1-175-2020

**Published:** 2020-08-07

**Authors:** Laurynas Dagys, Barbara Ripka, Markus Leutzsch, Gamal A. I. Moustafa, James Eills, Johannes F. P. Colell, Malcolm H. Levitt

**Affiliations:** 1 School of Chemistry, University of Southampton, Southampton SO17 1BJ, UK; 2 Max-Planck-Institut für Kohlenforschung, Kaiser-Wilhelm-Platz 1, 45470 Mülheim an der Ruhr, Germany; 3 Helmholtz Institute Mainz, Johannes Gutenberg University, 55099 Mainz, Germany

## Abstract

In the majority of hydrogenative parahydrogen-induced polarization (PHIP) experiments, the hydrogen molecule undergoes pairwise *cis* addition to an unsaturated precursor to occupy vicinal positions on the product molecule. However, some ruthenium-based hydrogenation catalysts induce *geminal* hydrogenation, leading to a reaction product in which the two hydrogen atoms are transferred to the same carbon centre, forming a methylene ( ${\mathrm{CH}}_{2}$ ) group. The singlet order of parahydrogen is substantially retained over the *geminal* hydrogenation reaction, giving rise to a singlet-hyperpolarized ${\mathrm{CH}}_{2}$ group. Although the $T_{1}$ relaxation times of the methylene protons are often short, the singlet order has a long lifetime, provided that singlet–triplet mixing is suppressed, either by chemical equivalence of the protons or by applying a resonant radiofrequency field. The long lifetime of the singlet order enables the accumulation of hyperpolarization during the slow hydrogenation reaction. We introduce a kinetic model for the behaviour of the observed hyperpolarized signals, including both the chemical kinetics and the spin dynamics of the reacting molecules. Our work demonstrates the feasibility of producing singlet-hyperpolarized methylene moieties by parahydrogen-induced polarization. This potentially extends the range of molecular agents which may be generated in a hyperpolarized state by chemical reactions of parahydrogen.

## 1 Introduction

Nuclear magnetic resonance (NMR) suffers from intrinsically low sensitivity, in part because of the small interaction energies between nuclear spins and magnetic fields. Hyperpolarization techniques alleviate this problem by generating nuclear spin systems with a high degree of nuclear spin polarization, enhancing the nuclear magnetization by many orders of magnitude ([Bibr bib1.bibx2]). Parahydrogen-induced polarization (PHIP) ([Bibr bib1.bibx5]) is a hyperpolarization method which utilizes hydrogen ( $H_{2}$ ) gas enriched in the *para*-spin isomer; the enrichment is carried out by cooling $H_{2}$ gas over a suitable catalyst. There are two main modes of PHIP: (i) in *hydrogenative* PHIP, the strongly enhanced nuclear singlet order of *para*-enriched $H_{2}$ gas is substantially conserved through a pairwise catalytic transfer of the hydrogen pair onto a product molecule ([Bibr bib1.bibx5]). The high degree of nuclear singlet order in the hydrogenation product is converted into enhanced nuclear magnetization by symmetry-breaking nuclear spin interactions; (ii) in the signal amplification by reversible exchange (SABRE) method, reversible chemical processes are used to transfer the nuclear singlet order onto the target molecules ([Bibr bib1.bibx1]). PHIP has several advantages over alternative hyperpolarization techniques, such as its low cost, its compact and simple equipment requirements, and its ability to produce relatively large amounts of hyperpolarized material in a short time.

This article concerns hydrogenative PHIP experiments, which involve in most cases the *vicinal* positioning of the hydrogen substituents; i.e. the hydrogen atoms become attached to *adjacent* carbon atoms in the product molecule. Furthermore, in the case that a carbon–carbon triple bond is hydrogenated, the hydrogenation product usually has the *cis* geometry; i.e. the two hydrogen atoms end up on the same side of the resulting double bond. This reaction specificity strongly limits the range of hyperpolarized substances accessible to hydrogenative PHIP.

Recent advances in catalytic chemistry have uncovered alternative modes of hydrogenation ([Bibr bib1.bibx16]). For example, some ruthenium-based catalysts achieve *trans*-vicinal hydrogenation, meaning that the two hydrogen atoms are transferred to *opposite* sides of the resulting double bond ([Bibr bib1.bibx22]). This phenomenon allows the hyperpolarization of the important metabolite fumarate in aqueous solution ([Bibr bib1.bibx32]). Furthermore, under some circumstances, *geminal* hydrogenation is observed, meaning that the two hydrogen atoms become bonded to the *same* carbon in the product molecule ([Bibr bib1.bibx15]). If *para*-enriched $H_{2}$ is used, the result is a methylene ( ${\mathrm{CH}}_{2}$ ) moiety in which the proton pair exhibits strongly enhanced nuclear singlet order, meaning a population difference between the nuclear singlet and triplet states ([Bibr bib1.bibx8]). If the product molecule has sufficiently low symmetry, the ${\mathrm{CH}}_{2}$ protons are magnetically inequivalent, allowing symmetry-breaking spin interactions to convert the nuclear singlet order into hyperpolarized nuclear magnetization. Since methylene groups are ubiquitous in metabolites and natural products, *gem*-PHIP could potentially open up a new range of PHIP-based hyperpolarization targets.

One difficulty with *gem*-PHIP is that the associated hydrogenation reaction is usually slow ([Bibr bib1.bibx34]). Furthermore, the short internuclear distance between the ${\mathrm{CH}}_{2}$ protons leads to a strong dipole–dipole interaction, which provides an efficient $T_{1}$ mechanism and hence rapid decay of hyperpolarized magnetization. The combination of a slow production rate of spin order with a short relaxation time $T_{1}$ leads to weak hyperpolarization, with poor enhancement factors and low polarization levels.

Although the $T_{1}$ values of methylene protons are usually short, their singlet relaxation times $T_{\text{S}}$ can be long, exceeding 2 min in some cases ([Bibr bib1.bibx6]). In most cases, these long singlet lifetimes are not immediately manifest, since symmetry-breaking interactions such as chemical shift differences between the ${\mathrm{CH}}_{2}$ protons mix the long-lived singlet state with the rapidly relaxing triplet states. Experimental intervention is usually needed to suppress singlet–triplet mixing, either by transferring the sample to a low magnetic field ([Bibr bib1.bibx8]) or by applying a resonant radiofrequency (rf) field ([Bibr bib1.bibx6]).

In this article we investigate the accumulation of a long-lived hyperpolarized singlet order on methylene protons during a *gem*-PHIP experiment by application of a spin-locking rf field during the slow chemical reaction ([Bibr bib1.bibx18]). We introduce a kinetic model to describe the observed hyperpolarization levels during experiments and provide a theoretical analysis of the spin dynamics.

## 2 *Geminal* hydrogenation

The *geminal* hydrogenation reaction studied in this paper is shown in Fig. [Fig Ch1.F1]. It involves the hydrogenation of acetylenedicarboxylate (top left), catalysed by the ruthenium complex $[{\mathrm{Cp}}^{*}\mathrm{Ru}({\mathrm{CH}}_{3}\mathrm{CN}{)}_{3}]{\mathrm{PF}}_{6}$ in $D_{2}O$ solution. The main product of this reaction is the *trans*-vicinal hydrogenation product, fumarate ([Bibr bib1.bibx32]) (see Appendix B). However, in some conditions, the side product **I** is also formed (the systematic name for **I** and an NMR spectrum of the reaction mixture are given in the Supplement). The side reaction is inhibited by sodium sulfite ([Bibr bib1.bibx32]). In the current work, sodium sulfite was not used, favouring generation of the *geminal* hydrogenation product **I**. The postulated reaction mechanism involves formation of a carbene intermediate ([Bibr bib1.bibx15]) between the catalyst and first acetylenedicarboxylate molecule, followed by a [ 3+2 ] cycloaddition with a second acetylenedicarboxylate molecule, dissociation of the ruthenium adduct, and abstraction of a deuterium atom from the $D_{2}O$ solvent.

**Figure 1 Ch1.F1:**
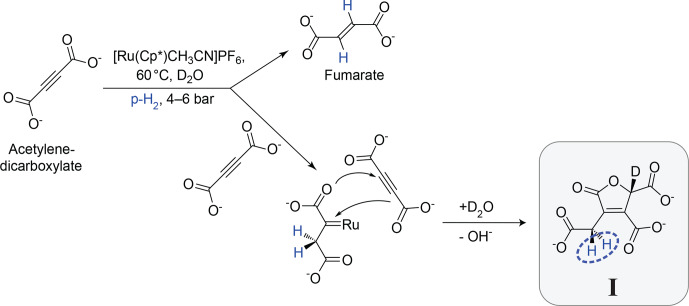
Postulated mechanism for the formation of **I**. The main hydrogenation reaction leads to the product fumarate. A side reaction, involving a second acetylenedicarboxylate molecule, leads to the product **I**. The inequivalent methylene ( ${\mathrm{CH}}_{2}$ ) protons which derive from *para*-enriched hydrogen are shown in blue.

**I** is prone to decomposition and further reactions and could not be isolated and subjected to standard characterization methods. As described in the Supplement, the structure of **I** was verified by synthesizing a compound with the same structure by an alternative route, followed by a comparison of the ${}^{1}H$ NMR spectra.

This paper focuses on the two ${\mathrm{CH}}_{2}$ protons of the product molecule **I** which derive from the *para*-enriched $H_{2}$ reagent. This proton pair is highlighted in blue in Fig. [Fig Ch1.F1]. The chemical equivalence of these ${\mathrm{CH}}_{2}$ protons is broken by the chiral centre four bonds away, on the opposite side of the five-membered ring.

Figure [Fig Ch1.F2] shows the ${\mathrm{CH}}_{2}$ region of the ${}^{1}H$ NMR spectrum of the reaction product. The black spectrum is the Fourier transform of the NMR signal induced by a single π/2 pulse, obtained 90 s after the conclusion of the chemical reaction with *para*-enriched hydrogen. It displays a typical AB pattern, albeit with some spectral intensity distortions from residual hyperpolarization effects (see Appendix B for further explanation). The two protons have a chemical shift difference of Δδ=0.197 ppm and a two-bond J coupling of ${|}^{2}J|=16.8$ Hz.

**Figure 2 Ch1.F2:**
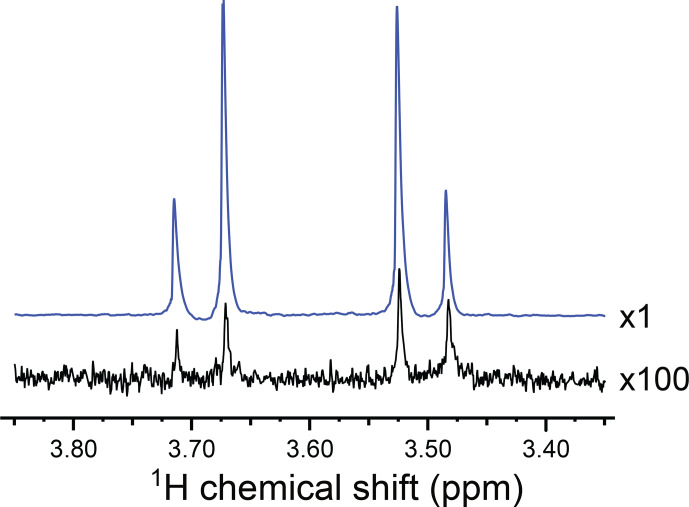
Partial ${}^{1}H$ NMR spectra of the reaction products at a resonance frequency of 400.0 MHz and temperature of 333 K, showing the signals from the ${\mathrm{CH}}_{2}$ group of **I**. The hyperpolarized spectrum (blue) was acquired in a *gem*-PHIP experiment using the pulse sequence in Fig. [Fig Ch1.F3]a, with the intervals $\tau_{1}=90$ s and $\tau_{2}=90$ s. The spectrum in black was obtained by waiting 90 s after the conclusion of the experiment and taking the Fourier transform of the NMR signal induced by a single π/2 pulse. The spectrum shows an AB peak pattern, with intensity distortions from residual hyperpolarization. The AB spectrum is consistent with a chemical shift difference of Δδ=0.197 ppm and a two-bond J coupling of ${|}^{2}J|=16.8$ Hz. The signal enhancement factor in the *gem*-PHIP experiment is estimated to be ∼300 , which corresponds to a ${}^{1}H$ polarization level of ∼0.9 %.

The nuclear spin relaxation characteristics of **I** were estimated at room temperature (295 K) and a magnetic field of 9.4 T, using standard techniques (see Supplement). The spin-lattice relaxation time of the ${\mathrm{CH}}_{2}$ protons is given by $T_{1}=1.23\pm 0.14$ s. The singlet relaxation time of the ${\mathrm{CH}}_{2}$ protons under the same conditions is $T_{\text{S}}=61.1\pm 7.1$ s. Unfortunately, the chemical instability of **I** made it difficult to estimate the relaxation times under the much warmer conditions of the *gem*-PHIP reaction (333 K; see Sect. [Sec Ch1.S5]). As described in the Supplement, molecule **I** decomposes by losing one carboxylic acid group to give an achiral reaction product. The decomposition occurs on a timescale of roughly 2 h at 333 K.

## 3 Results

### 3.1 *gem*-PHIP

Parahydrogen-induced hyperpolarization of **I** was demonstrated using the pulse sequence in Fig. [Fig Ch1.F3]a. Bubbling of *para*-enriched hydrogen was conducted for an interval $\tau_{1}=90$ s in the presence of a radiofrequency spin-locking field ([Bibr bib1.bibx18]), whose frequency corresponds to the mean chemical shift of the ${\mathrm{CH}}_{2}$ protons. The spin-locking field amplitude corresponded to a ${}^{1}H$ nutation frequency of $\omega_{\mathrm{nut}}/2\pi =1.0$ kHz. Bubbling was switched off and the spin locking continued for a further interval of $\tau_{2}=30$ s. This gave time for the bubbles to dissipate and for a hyperpolarized singlet order to accumulate during the ongoing hydrogenation reaction.

**Figure 3 Ch1.F3:**
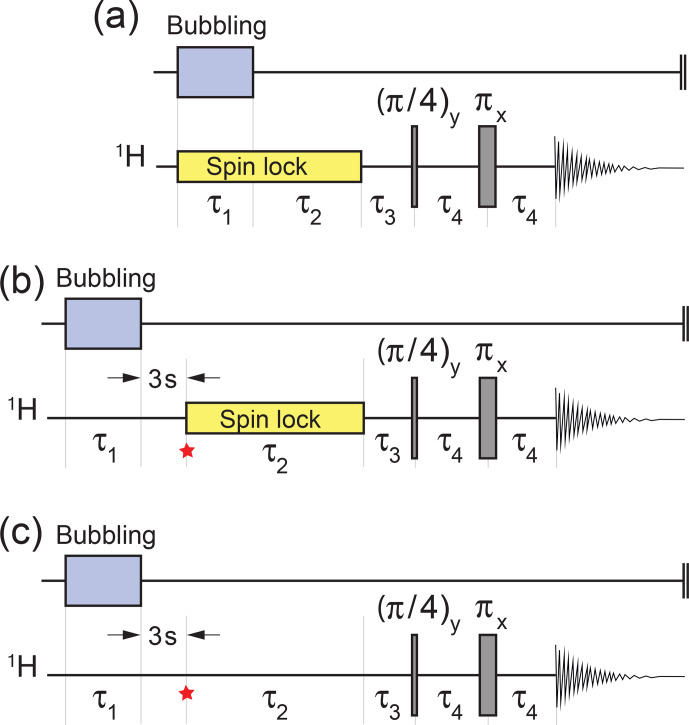
Experimental timing sequences. **(a)** Procedure for the demonstration of *gem*-PHIP. Bubbling of *para*-enriched $H_{2}$ is conducted for an interval $\tau_{1}$ in the presence of a radio-frequency spin-locking field in order to suppress singlet–triplet mixing in the reaction product **I**. The spin locking continues for a further interval $\tau_{2}$ , followed by a two-pulse sequence to convert the hyperpolarized singlet order to in-phase magnetization ([Bibr bib1.bibx33]). The experimental delays were $\tau_{3}=6.49$ ms and $\tau_{4}=14.97$ ms. **(b)** Procedure for demonstrating the accumulation of the singlet order during spin locking. The spin-lock field is applied during the variable interval $\tau_{2}$ , with an amplitude corresponding to a nutation frequency $\omega_{\mathrm{nut}}/(2\pi )=1.0$ kHz. The star symbol refers to the time point discussed in the text. **(c)** The same sequence as for panel **(b)** but without spin locking during the variable $\tau_{2}$ interval. The interval $\tau_{1}$ was set to 90 s for panel **(a)** and 17 s for panels **(b)** and **(c)**.

Hyperpolarized singlet order was converted into in-phase magnetization by the sequence of three delays and two radiofrequency pulses shown in Fig. [Fig Ch1.F3]. This sequence converts magnetization into singlet order in weakly coupled spin- 1/2 pairs ([Bibr bib1.bibx33]). The ideal values of the pulse sequence delays, in the case of infinitely short pulses, are $\tau_{3}=|\pi /\omega_{\Delta }|$ and $\tau_{4}=1/(4J)$ , where the chemical shift frequency difference is $\omega_{\Delta }=\omega^{0}\Delta \delta$ and $\omega^{0}$ is the Larmor frequency. In practice, the following pulse sequence intervals were used: $\tau_{3}=6.49$ ms and $\tau_{4}=14.97$ ms.

Figure [Fig Ch1.F2] shows the ${}^{1}H$ NMR spectrum of **I**, hyperpolarized by *gem*-PHIP (blue spectrum). The NMR signals of the ${\mathrm{CH}}_{2}$ protons are enhanced by a factor of ∼300 as compared to the spectrum taken 90 s after the end of the pulse sequence (black spectrum). This enhancement factor corresponds to a modest polarization level of ∼0.9 %. Although the achieved polarization level is not spectacular, this experiment demonstrates the feasibility of the *gem*-PHIP of methylene protons, provided that a spin-locking field is used to stabilize the hyperpolarized singlet order.

### 3.2 Hyperpolarization decay

Figure [Fig Ch1.F4] shows the dependence of the integrated *gem*-PHIP signal intensity on the spin-locking interval $\tau_{2}$ in Fig. [Fig Ch1.F3]a, with the bubbling time $\tau_{1}$ increased to 90 s. Each point in Fig. [Fig Ch1.F4] is the result of an independent experiment, performed on a fresh aliquot of the stock solution, with the signal amplitude normalized against the integrated amplitude of the thermal equilibrium spectrum, obtained 90 s after the pulse sequence has finished. The integrated signal amplitude follows a monoexponential decay function with a time constant of 151±9 s. As discussed below, this time constant may be assigned to the decay time constant $T_{\text{S}}$ for a singlet order in the presence of the spin-locking field.

**Figure 4 Ch1.F4:**
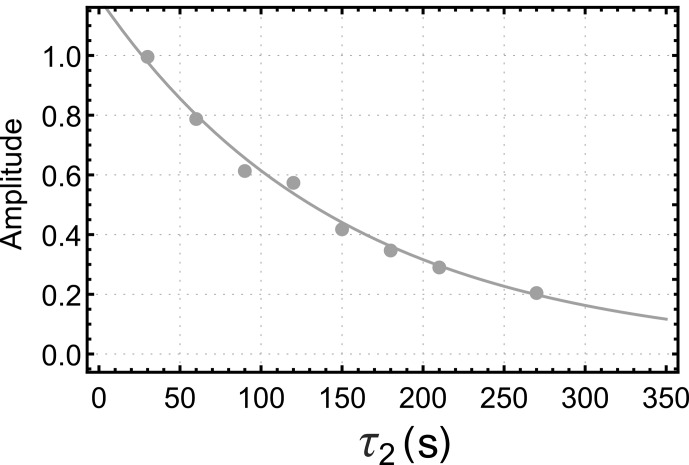
Dependence of the integrated *gem*-PHIP signal amplitude for the ${\mathrm{CH}}_{2}$ protons of **I** on the interval $\tau_{2}$ in the pulse sequence of Fig. [Fig Ch1.F3]a, with $\tau_{1}$ fixed to 90 s. Solid line: fit to Eq. ([Disp-formula Ch1.E19]) with $f_{a}A_{a}=1.79$ , $f_{a}B_{a}=0$ and time constant $T_{S}^{\text{I}}=151$ s for singlet-order decay.

### 3.3 Accumulation of a hyperpolarized singlet order

The pulse sequence in Fig. [Fig Ch1.F3]b was used to study the accumulation of a hyperpolarized singlet order on the ${\mathrm{CH}}_{2}$ protons of **I** during the slow *geminal* hydrogenation reaction. The experiment started by bubbling *para*-enriched $H_{2}$ gas through the NMR tube for $\tau_{1}=17$ s, in order to saturate the solution. The sample was then allowed to rest for a settling time of 3 s in order to dissipate bubbles and to achieve good sample and field homogeneity. The trajectory of the hyperpolarized spin order during the subsequent interval was followed by varying the interval $\tau_{2}$ in a series of independent experiments, each one performed on a separate aliquot of the same stock solution. Experiments were also performed without spin locking during the $\tau_{2}$ interval (Fig. [Fig Ch1.F3]c).

The results of this investigation are shown in Fig. [Fig Ch1.F5]. When a spin-locking field is applied during the $\tau_{2}$ interval (Fig. [Fig Ch1.F3]b), the hyperpolarized signals first increase and then decay (orange symbols). If no spin-locking field is applied during the $\tau_{2}$ interval (Fig. [Fig Ch1.F3]c), the hyperpolarized NMR signals decay monotonically with respect to $\tau_{2}$ (blue symbols).

**Figure 5 Ch1.F5:**
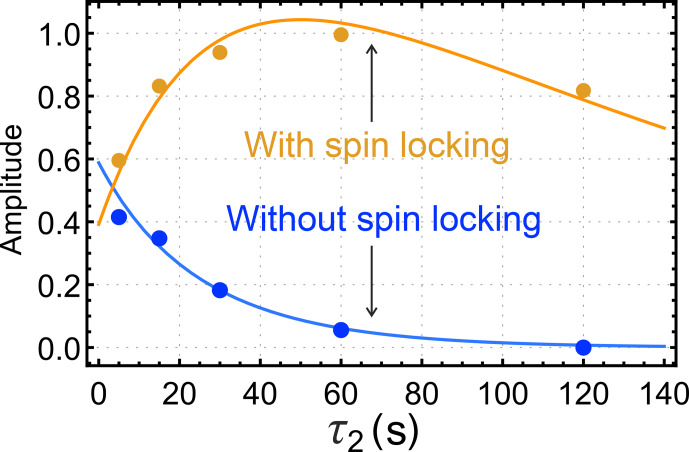
Dependence of the integrated *gem*-PHIP signal amplitudes of the ${\mathrm{CH}}_{2}$ protons of **I** on the interval $\tau_{2}$ in the pulse sequences of Fig. [Fig Ch1.F3]b and c, with $\tau_{1}$ fixed to 17 s. The orange symbols show the amplitudes for the case of a spin-locking field during the $\tau_{2}$ interval (sequence in Fig. [Fig Ch1.F3]b), with an amplitude corresponding to the nutation frequency $\omega_{\mathrm{nut}}/2\pi =1.0$ kHz. The blue symbols show the amplitudes for experiments without a spin-locking field during the $\tau_{2}$ interval (sequence in Fig. [Fig Ch1.F3]c). The orange and blue solid lines show the functions $a_{b}(\tau_{2})$ and $a_{c}(\tau_{2})$ (Eqs. [Disp-formula Ch1.E19] and [Disp-formula Ch1.E23], respectively), with the parameters $T_{S}^{\text{I}}=151$ s, $T_{\Sigma }^{H_{2}}=28.7$ s, $T_{zz}^{\text{I}}=13.2$ s, $f_{b}C_{\text{SO}}^{H_{2}}(0)\times k=0.059\;s^{-1}$ , $f_{c}C_{zz}^{\text{I}}(0)=-1.2$ , and $f_{b}=f_{c}$ .

## 4 Kinetic analysis

Figure [Fig Ch1.F6] shows the simplified kinetic model which is used to interpret these results. The dynamics of the system may be analysed in terms of the chemical kinetics of the hydrogenation reaction as well as the spin dynamics of the product molecule **I**. Although the chemical kinetics depend only on concentrations and physical conditions, the spin-dynamical pathways may be manipulated experimentally with fine time resolution, for example by turning spin-locking fields on and off. The experimental results derive from an interplay between the chemical and spin-dynamical domains. Similar analyses have been performed in different contexts ([Bibr bib1.bibx19]).

**Figure 6 Ch1.F6:**
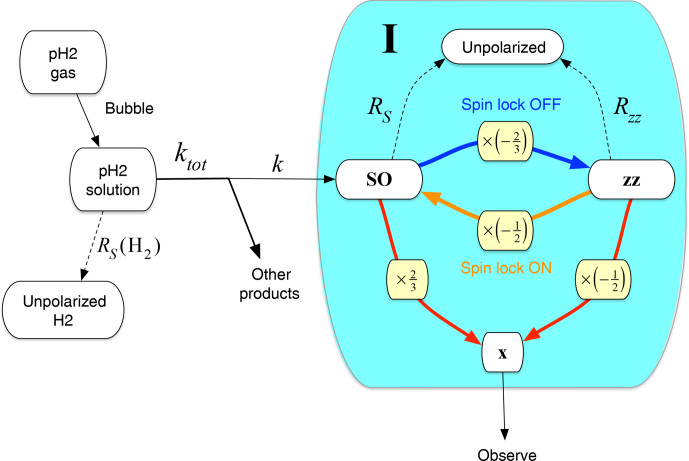
Kinetic model for *gem*-PHIP. The chemical reaction of *para*-enriched $H_{2}$ with the acetylenedicarboxylate precursor and ruthenium-based catalyst (not shown) proceeds with rate constant k . The shaded area includes the product molecule **I** in different spin polarization states: unpolarized (top), in a state of singlet nuclear spin order (left), in a state of zz order (right), and with observable x magnetization (bottom). Singlet order decays with rate constant $R_{S}=T_{\text{S}}^{-1}$ ; zz order decays with rate constant $R_{zz}=T_{zz}^{-1}$ . If no spin-locking field is present, singlet order is rapidly converted into zz order, with a conversion factor of -2/3 (blue arrow and yellow box). If a spin-locking field is applied, zz order is instantaneously projected onto singlet order, with a conversion factor of -1/2 (orange arrow and yellow box). Singlet order and zz order may be converted into observable x magnetization by the two-pulse sequence in Fig. [Fig Ch1.F3] (red arrows and yellow boxes). The conversion factors in this case are 2/3 for singlet order and -1/2 for zz order.

### 4.1 Chemical kinetics

After *para*-enriched $H_{2}$ gas is introduced into the solution by bubbling, it starts to react with the acetylenedicarboxylate precursor, catalysed by the ruthenium complex. As depicted in Fig. [Fig Ch1.F1], this is a complex process with the generation of several products and with the production of **I** requiring an additional precursor molecule. Nevertheless, for the sake of simplicity, and since the acetylenedicarboxylate precursor is in excess, the reaction leading to **I** is assumed to be first order with respect to the *para*- $H_{2}$ reagent and to proceed with rate constant k .

After the bubbling has stopped, the concentrations of the $H_{2}$ reagent and the product molecule **I** are assumed to follow the simple kinetic equations:

1 $$\begin{array}{rl} & \frac{d}{dt}{\left[H_{2}\right]}_{t}=-k_{\mathrm{tot}}{\left[H_{2}\right]}_{t},\\ & \frac{d}{dt}{\left[\text{I}\right]}_{t}=+k{\left[H_{2}\right]}_{t}, \end{array}$$

where $k_{\mathrm{tot}}$ is the rate constant for all hydrogenation reactions, including those that do not lead to the product molecule **I**, $k_{\mathrm{tot}}>k$ . The differential Eq. ([Disp-formula Ch1.E1]) is easily solved to show that the solution concentration of $H_{2}$ decays exponentially with time, while the concentration of the product molecule **I** increases:

2H2t=H20exp⁡(-ktott),3It=I∞1-exp⁡(-kt),

where the limiting value of the concentration of **I** is given by

4 $${\left[\text{I}\right]}_{\infty }=\frac{k}{k_{\mathrm{tot}}}{\left[H_{2}\right]}_{0}.$$

As expected, the limiting yield of **I** depends on the ratio of the productive rate constant k to the total rate constant of all hydrogenation reactions $k_{\mathrm{tot}}$ .

### 4.2 Spin dynamics

In this section we discuss some general spin-dynamical scenarios relevant to parahydrogen-enhanced NMR experiments, and in Sect. [Sec Ch1.S4.SS3] we evaluate the spin dynamics and chemical kinetics specific to the experiments performed in this work. The spin dynamics in this section were evaluated with the assistance of *SpinDynamica* software ([Bibr bib1.bibx4]).

#### 4.2.1 Singlet order

The proton spins of *para*-enriched $H_{2}$ are in a state of enhanced nuclear singlet order, described as the difference between the population of the nuclear singlet state and the mean population of the nuclear triplet states:

5 $$\mathrm{SO}=\langle S_{0}|\rho |S_{0}\rangle -\frac{1}{3}\sum_{M=-1}^{+1}\langle T_{M}|\rho |T_{M}\rangle ,$$

where ρ is the spin density operator, and the singlet and triplet states are defined in terms of the Zeeman product states as follows ([Bibr bib1.bibx23]):

6 $$\begin{array}{rl} & |S_{0}\rangle =2^{-1/2}\left(|\alpha \beta \rangle -|\beta \alpha \rangle \right),\\ & |T_{+1}\rangle =|\alpha \alpha \rangle ,\\ & |T_{0}\rangle =2^{-1/2}\left(|\alpha \beta \rangle +|\beta \alpha \rangle \right),\\ & |T_{-1}\rangle =|\beta \beta \rangle . \end{array}$$

Singlet order SO may be regarded as the expectation value of the singlet order operator $Q_{\mathrm{SO}}$ , which is defined as follows:

7 $$Q_{\mathrm{SO}}=|S_{0}\rangle \langle S_{0}|-\frac{1}{3}\sum_{M=-1}^{+1}|T_{M}\rangle \langle T_{M}|=-\frac{4}{3}I_{1}\cdot I_{2},$$

so that

8 $$\mathrm{SO}=\langle Q_{\mathrm{SO}}\rangle =\mathrm{Tr}\;\left\{Q_{\mathrm{SO}}^{†}\rho \right\}.$$

$H_{2}$ gas in thermal equilibrium at room temperature, with an *ortho* : *para* ratio very close to 3 : 1, has a negligible singlet order, SO≃0 . Pure *para*- $H_{2}$ gas has singlet order SO=1 . The current work employs $H_{2}$ gas which is enriched with the *para*-spin isomer by thermal equilibration at 77 K. This yields an *ortho* : *para* ratio of approximately 1 : 1, corresponding to a singlet order of SO≃1/3 . Assuming that the nuclear spin states are substantially unchanged through the chemical reaction, the product molecule **I** is formed with its methylene protons in a similar state of finite singlet order, SO≃1/3 .

The singlet-order operator $Q_{\mathrm{SO}}$ is an exact eigenoperator of the spin propagation superoperator in the case of a magnetically equivalent spin-pair system such as for $H_{2}$ gas. However, in the product molecule **I**, the chiral centre breaks the equivalence of the methylene protons, so that the operator $Q_{\mathrm{SO}}$ is no longer an eigenoperator of the evolution. The chemical shift difference induces singlet–triplet transitions which mix the operator $Q_{\mathrm{SO}}$ with other operators. However, if a sufficiently strong spin-locking field is applied, the singlet–triplet transitions are suppressed, so that the order SO is substantially unchanged during the evolution, except for a decay due to relaxation processes ([Bibr bib1.bibx28]). The decay rate constant is given by $R_{\text{S}}=T_{\text{S}}^{-1}$ , where $T_{\text{S}}$ is the time constant for singlet-order decay, which is often much longer than the relaxation time constant $T_{1}$ for longitudinal magnetization. The decay of singlet order in the presence of a spin-locking field, with rate constant $R_{\text{S}}$ , is shown in Fig. [Fig Ch1.F6] by the dashed arrow running upwards, connecting the SO state of the reaction product **I** to the unpolarized state.

#### 4.2.2 zz order

A different type of nuclear spin order is called zz order ([Bibr bib1.bibx35]) and corresponds to the expectation value of an operator $Q_{zz}$ defined as follows:

9 $$\begin{array}{rl} & Q_{zz}=2I_{1z}I_{2z},\\ & \mathrm{zz}=\langle Q_{zz}\rangle =\mathrm{Tr}\;\left\{Q_{zz}^{†}\rho \right\}. \end{array}$$

In the absence of a spin-locking field, and if there is a relatively large chemical shift difference between the coupled spins, the operator $Q_{zz}$ is a better approximation to an eigenoperator of the spin evolution propagator than the singlet-order operator $Q_{\mathrm{SO}}$ . The relaxation of the system can be complex and multi-exponential in this case. Nevertheless, for the sake of simplicity, we assume here a single rate constant $R_{zz}=T_{zz}^{-1}$ for the zz order in the absence of a spin-locking field. The time constant $T_{zz}$ is expected to be close to the ordinary spin-lattice relaxation time constant $T_{1}$ . The decay of zz order in the absence of a spin-locking field, with rate constant $R_{zz}$ , is shown in Fig. [Fig Ch1.F6] by the dashed arrow running upwards connecting the zz state of the reaction product **I** to the unpolarized state.

#### 4.2.3 Spin locking OFF

Suppose that the molecules of **I** are in a state of enhanced singlet order SO . This state is stable if a spin-locking field is continuously applied and decays monotonically with the time constant $T_{\text{S}}$ . However, if the spin-locking field is turned off, the chemical shift difference between the methylene protons leads to rapid singlet–triplet mixing. The zz -order operator $Q_{zz}$ is an approximate eigenoperator of the evolution in this case, instead of the singlet-order operator $Q_{\mathrm{SO}}$ . Hence, any singlet order SO which is present when the spin-locking field is turned off is projected onto the zz -order operator $Q_{zz}$ . The remaining spin order corresponds to zero-quantum coherences which rapidly oscillate and decay. These additional components may be ignored to a good approximation, provided that the spin-locking field remains turned off for an interval that is long compared to the difference in chemical shift frequencies.

The zz order created by this projection process is given by

10 $$\mathrm{zz}=\frac{\mathrm{Tr}\;\left\{Q_{zz}^{†}Q_{\mathrm{SO}}\right\}}{\mathrm{Tr}\;\left\{Q_{zz}^{†}Q_{zz}\right\}}\mathrm{SO}=-\frac{2}{3}\mathrm{SO}.$$

The projection of SO onto zz is depicted by the blue arrow in Fig. [Fig Ch1.F6], annotated by the projection factor -2/3 (yellow box).

#### 4.2.4 Spin locking ON

Suppose that the molecules of **I** are in a state of enhanced zz order zz . The corresponding operator $Q_{zz}$ is an eigenoperator of the spin evolution in the absence of a spin-locking field. However, if the spin-locking field is turned on, singlet–triplet mixing is suppressed, and the zz -order operator $Q_{zz}$ is no longer an eigenoperator of the spin evolution. Any zz order which is present when the spin-locking field is turned on is projected onto the singlet-order operator $Q_{\mathrm{SO}}$ . The remaining spin order corresponds to high-rank spin order terms which rapidly dephase under radiofrequency field inhomogeneity.

The singlet order created by this projection process is given by

11 $$\mathrm{SO}=\frac{\mathrm{Tr}\;\left\{Q_{\mathrm{SO}}^{†}Q_{zz}\right\}}{\mathrm{Tr}\;\left\{Q_{\mathrm{SO}}^{†}Q_{\mathrm{SO}}\right\}}\mathrm{zz}=-\frac{1}{2}\mathrm{zz}.$$

The projection of zz onto SO is depicted by the orange arrow in Fig. [Fig Ch1.F6], annotated by the projection factor -1/2 (yellow box).

#### 4.2.5 Signal read-out

The spin orders zz and SO are observed by applying the two-pulse sequence given in Fig. [Fig Ch1.F3]c and described in [Bibr bib1.bibx33]. This sequence converts both types of spin order into observable transverse magnetization, which induces a time-domain NMR signal in the subsequent interval of free precession. The read-out transformations may be written as follows:

12 $$\begin{array}{rl} & UQ_{\mathrm{SO}}U^{†}=a(\mathrm{SO}\to x)I_{x}+\ldots ,\\ & UQ_{zz}U^{†}=a(\mathrm{zz}\to x)I_{x}+\ldots , \end{array}$$

where U is the propagator for the two-pulse sequence and the dots denote operators which are orthogonal to $I_{x}$ . These amplitudes may be calculated as follows:

13 $$\begin{array}{rl} & a(\mathrm{SO}\to x)=\frac{\mathrm{Tr}\;\left\{I_{x}^{†}UQ_{\mathrm{SO}}U^{†}\right\}}{\mathrm{Tr}\;\left\{I_{x}^{2}\right\}},\\ & a(\mathrm{zz}\to x)=\frac{\mathrm{Tr}\;\left\{I_{x}^{†}UQ_{zz}U^{†}\right\}}{\mathrm{Tr}\;\left\{I_{x}^{2}\right\}}. \end{array}$$

In an ideal weakly coupled spin system, with infinitely short, ideal, radiofrequency pulses and delays given by $\tau_{3}=|\pi /\omega_{\Delta }|$ and $\tau_{4}=1/(4J)$ , the transformation amplitudes are as follows:

14 $$\begin{array}{rl} & a(\mathrm{SO}\to x)=\frac{2}{3},\\ & a(\mathrm{zz}\to x)=-\frac{1}{2}. \end{array}$$

These transformations are indicated by the red arrows and yellow boxes in Fig. [Fig Ch1.F6].

The integrated amplitude of the NMR spectrum obtained by Fourier transformation of the NMR signal is therefore proportional to the zz and SO orders before the read-out sequence is applied, multiplied by the transformation factors in Eq. ([Disp-formula Ch1.E14]).

### 4.3 Analysis of experimental trajectories

The chemical kinetics and spin dynamics may be combined to achieve an understanding of the trajectories in Figs. [Fig Ch1.F4] and [Fig Ch1.F5] generated by the timing sequences shown in Fig. [Fig Ch1.F3].

#### 4.3.1 Trajectories with spin locking

The pulse sequences in Fig. [Fig Ch1.F3]a, b both examine the dependence of hyperpolarized signals on the duration $\tau_{2}$ of a spin-locking interval. However, the state of the spin system at the start of the $\tau_{2}$ interval is different in the two procedures. In Fig. [Fig Ch1.F3]a, which provides the results shown in Fig. [Fig Ch1.F4], spin locking is applied continuously during the bubbling interval and continued during the variable delay $\tau_{2}$ . In the sequence of Fig. [Fig Ch1.F3]b, on the other hand, which provides the orange data points in Fig. [Fig Ch1.F5], the spin locking is interrupted for 3 s before the $\tau_{2}$ interval starts.

In both cases, the evolution of the singlet order during the spin-locking interval obeys the following differential equations:

15 $$\begin{array}{rl} & \frac{d}{dt}C_{\text{SO}}^{H_{2}}(t)=-R_{\Sigma }^{H_{2}}C_{\text{SO}}^{H_{2}}(t),\\ & \frac{d}{dt}C_{\text{SO}}^{\text{I}}(t)=+kC_{\text{SO}}^{H_{2}}(t)-R_{S}^{\text{I}}C_{\text{SO}}^{\text{I}}(t), \end{array}$$

The notation $C_{\text{SO}}^{X}(t)$ indicates the total amplitude of singlet spin order for the species X at time point t , taking into account the concentration of X as well as its spin state. The decay rate constant for singlet order in compound **I**, due to spin-dynamical processes, is denoted $R_{S}^{\text{I}}=T_{\text{S}}(\text{I}{)}^{-1}$ . The total decay rate constant for $H_{2}$ singlet order, due to the combination of chemical and spin-dynamical processes, is denoted

16 $$R_{\Sigma }^{H_{2}}=k_{\mathrm{tot}}+R_{S}^{H_{2}},$$

where $R_{S}^{H_{2}}$ denotes the decay rate constant for $H_{2}$ singlet order, due to *para*-to-*ortho* conversion in solution, in the presence of the hydrogenation catalyst but in the absence of a hydrogenation reaction. Note that this rate constant may be greatly increased by the presence of the catalyst, since transient binding of $H_{2}$ molecules with the catalyst provides an efficient mechanism for *ortho*–*para* conversion.

Equation ([Disp-formula Ch1.E15]) may be solved to obtain the following trajectory of the singlet order for compound **I** under spin locking:

17 $$C_{\text{SO}}^{\text{I}}(\tau_{2},i)=A_{i}\mathrm{exp}\left\{-R_{S}^{\text{I}}\tau_{2}\right\}+B_{i}\mathrm{exp}\left\{-R_{\Sigma }^{H_{2}}\tau_{2}\right\},$$

where the coefficients are

18 $$\begin{array}{rl} & A_{i}=C_{\text{SO}}^{\text{I}}(0,i)-B_{i},\\ & B_{i}=\frac{kC_{\text{SO}}^{H_{2}}(0,i)}{R_{S}^{\text{I}}-R_{\Sigma }^{H_{2}}}. \end{array}$$

The index i refers to the first two pulse sequences in Fig. [Fig Ch1.F3], i∈{a,b} . The symbol $C_{\text{SO}}^{\text{I}}(0,i)$ is the total amplitude of $H_{2}$ singlet order at the start of the spin-lock interval in experiment i , taking into account the concentration of **I** as well as its spin state.

The amplitude factor for the read-out of a singlet order is given by (+2/3) , as shown by Eq. ([Disp-formula Ch1.E14]). Hence the integrated signal amplitudes for the sequences in Fig. [Fig Ch1.F3]a, b are given by

19 $$a_{i}(\tau_{2})=\frac{2}{3}f_{i}\left(A_{i}\mathrm{exp}\left\{-R_{S}^{\text{I}}\tau_{2}\right\}+B_{i}\mathrm{exp}\left\{-R_{\Sigma }^{H_{2}}\tau_{2}\right\}\right),$$

where $f_{i}$ are instrumental factors and i∈{a,b} . The signal trajectories have a bi-exponential form, in general.

#### 4.3.2 Trajectory without spin locking

The sequence in Fig. [Fig Ch1.F3]c is identical to that in Fig. [Fig Ch1.F3]b, except for the absence of the spin-locking field during the $\tau_{2}$ interval. In the absence of spin locking, the relevant eigenoperator of the spin evolution during the $\tau_{2}$ interval is the zz operator $Q_{zz}$ (Eq. [Disp-formula Ch1.E9]). The combined chemical–spin dynamics of the system is described by the following differential equations:

20 $$\begin{array}{rl} & \frac{d}{dt}C_{\text{SO}}^{H_{2}}(t)=-R_{\Sigma }^{H_{2}}C_{\text{SO}}^{H_{2}}(t),\\ & \frac{d}{dt}C_{zz}^{\text{I}}(t)=(-\frac{2}{3})kC_{\text{SO}}^{H_{2}}(t)-R_{zz}^{\text{I}}C_{zz}^{\text{I}}(t), \end{array}$$

The factor (-2/3) appears since the $H_{2}$ singlet order is projected onto the zz order of the product molecule **I** upon hydrogenation, as described in Sect. [Sec Ch1.S4.SS2.SSS3]. This equation is valid provided that the interval t exceeds the time required for dephasing of spin-order components orthogonal to zz order after hydrogenation in the absence of a spin-locking field.

The differential Eq. ([Disp-formula Ch1.E20]) may be solved to obtain the following trajectory for the zz order of compound **I**, under the pulse sequence of Fig. [Fig Ch1.F3]c:

21 $$C_{zz}^{\text{I}}(t,c)=A_{c}\mathrm{exp}\left\{-R_{zz}^{\text{I}}t\right\}+B_{c}\mathrm{exp}\left\{-R_{\Sigma }^{H_{2}}t\right\},$$

where the coefficients are

22 $$\begin{array}{rl} & A_{c}=C_{zz}^{\text{I}}(0,c)-B_{c},\\ & B_{c}=-\frac{2kC_{\text{SO}}^{H_{2}}(0,c)}{3\left(R_{zz}^{\text{I}}-R_{\Sigma }^{H_{2}}\right)}. \end{array}$$

Here $C_{zz}^{\text{I}}(0,c)$ is the zz order of compound **I** at the beginning of the $\tau_{2}$ interval. This equation assumes that t is longer than the time required for dephasing of spin-order components orthogonal to the singlet order, in the presence of the spin-locking field. The zz order at the end of the $\tau_{2}$ interval is transformed into observable x magnetization by applying a sequence of two pulses and three delays. The amplitude factor for the read-out of zz order is given by (-1/2) , as shown by Eq. ([Disp-formula Ch1.E14]). Hence the integrated signal amplitude for the sequence in Fig. [Fig Ch1.F3]c is proportional to

23 $$a_{c}(\tau_{2})=-\frac{1}{2}f_{c}\left(A_{c}\mathrm{exp}\left\{-R_{zz}^{\text{I}}\tau_{2}\right\}+B_{c}\mathrm{exp}\left\{-R_{\Sigma }^{H_{2}}\tau_{2}\right\}\right).$$

This also has the form of a bi-exponential decay.

Since the sequences in Fig. [Fig Ch1.F3]b and c are the same up to the start of the $\tau_{2}$ interval (indicated by the star in the pulse-sequence diagrams), the instrumental factors are identical ( $f_{b}=f_{c}$ ) and we can write

24 $$C_{\text{SO}}^{\text{I}}(0,b)=-\frac{1}{2}C_{zz}^{\text{I}}(0,c)$$

using the projection in Eq. ([Disp-formula Ch1.E11]). Hence the signal amplitudes for these two experiments have the following relationship when extrapolated back to the start of the $\tau_{2}$ interval:

25 $$\frac{\alpha_{b}(0)}{\alpha_{c}(0)}=\frac{2}{3}.$$

The difference in extrapolated starting points is evident in the theoretical curves shown by the solid lines in Fig. [Fig Ch1.F5].

Since the procedures in Fig. [Fig Ch1.F3]b and c are identical for $\tau_{2}=0$ , one would expect $\alpha_{b}(0)=\alpha_{c}(0)$ , in contradiction to Eq. ([Disp-formula Ch1.E25]). This apparent paradox is resolved by noting that the derivation of Eq. ([Disp-formula Ch1.E25]) relies on the projections in Eqs. ([Disp-formula Ch1.E10]) and ([Disp-formula Ch1.E11]), which are not valid for very short intervals $\tau_{2}$ .

#### 4.3.3 Data fitting

The data sets of Figs. [Fig Ch1.F4] and [Fig Ch1.F5] were fitted simultaneously using the set of global parameters. All three data sets were well fitted by the functions $a_{a}(\tau_{2})$ , $a_{b}(\tau_{2})$ , and $a_{c}(\tau_{2})$ (Eqs. [Disp-formula Ch1.E19] and [Disp-formula Ch1.E23]) with the following parameters: $T_{S}^{\text{I}}=151\pm 9$ s; $T_{\Sigma }^{H_{2}}=28.7\pm 3.8$ s; $T_{zz}^{\text{I}}=13.2\pm 1.3$ s; $f_{a}A_{a}=1.79\pm 0.07$ ; $f_{b}B_{a}\approx 0$ ; $f_{b}C_{\text{SO}}^{H_{2}}(0,b)\times k=0.059\pm 0.007\;s^{-1}$ ; $f_{c}C_{zz}^{\text{I}}(0,c)=-1.2\pm 0.1$ . All rate constants are expressed here as time constants, i.e. $T_{X}=R_{X}^{-1}$ . The parameters $f_{b}C_{\text{SO}}^{H_{2}}(0,b)$ and k interact strongly in the fit and could not be determined independently. The coefficient of determination $R^{2}$ was estimated to be 0.991 for the fit in Fig. [Fig Ch1.F4] and 0.925 and 0.966 for the fits of the build-up and decay curves in Fig. [Fig Ch1.F5], respectively.

For these parameters, the trajectory in Fig. [Fig Ch1.F4] is very close to a single-exponential decay with time constant $T_{S}^{\text{I}}=151\pm 9$ s. For the case of the orange curve in Fig. [Fig Ch1.F5], on the other hand, the singlet order on **I** starts at a relatively low level. The long singlet decay time constant allows accumulation of a singlet order as the reaction proceeds in the presence of the spin-locking field. This accumulation gives rise to the rising initial trajectory of the orange curve in Fig. [Fig Ch1.F5]. The comparatively short time constant for the decay of zz order, $T_{zz}^{\text{I}}≃13.2$ s, allows no time for zz order to accumulate in the absence of a spin-locking field, giving rise to the monotonically decaying blue curve in Fig. [Fig Ch1.F5].

**Table 1 Ch1.T1:** Experimental procedure for gem-PHIP experiments.

Duration	Event
–	Inject 300 µ L of sample solution into the NMR tube.
1 min	Pressurize and bubble sample with inert gases at 4 bar. Depressurize.
10 min	Put sample in the magnet and raise temperature from 40 to 60 ${}^{\circ }$ C.
10 s	Pressurize sample with parahydrogen.
10 s	Bubble sample with parahydrogen to saturate sample and to pre-activate the catalyst.
5 min	Establish field homogeneity (shimming).
Variable	Perform the experiment.
–	Lower temperature to 40 ${}^{\circ }$ C and depressurize the sample.

The singlet decay time constant for the methylene protons of compound **I** was determined independently by non-hyperpolarized experiments (see Supplement). These experiments were performed at a much lower sample temperature of 295 K to avoid the decomposition of **I**. The estimated value of $T_{S}^{\text{I}}$ at 295 K and a magnetic field of 9.41 T is 61.1±7.1 s. This value is much shorter than the estimate of $T_{S}^{\text{I}}=151\pm 9$ s from the hyperpolarization trajectories. The discrepancy may be due in part to a reduction in rotational correlation time for the molecules of **I** at the elevated temperature used in the PHIP experiments.

## 5 Materials and methods

All experiments were conducted on a Bruker Avance Neo 400 MHz (9.41 T) system equipped with a 5 mm BBO probe. The excitation pulses were applied on-resonance with the doublet at 3.6 to 3.7 ppm. Their amplitude corresponded to a nutation frequency of ∼20 kHz. The spectral width was set to 20 ppm with sampling of 65k points.

The reagent solution consisted of 100 mM disodium acetylenedicarboxylate and 6 mM $[{\mathrm{Cp}}^{*}\mathrm{Ru}({\mathrm{CH}}_{3}\mathrm{CN}{)}_{3}]{\mathrm{PF}}_{6}$ dissolved in $D_{2}O$ . All sample solutions were prepared by mixing the components, sonicating the mixture for 5 min at 50 ${}^{\circ }$ C and filtering it through a 0.2 µ m pore-size syringe filter with a nylon membrane.

*Para*-enriched hydrogen was produced by slowly passing hydrogen gas through an iron oxide catalyst submerged in liquid nitrogen to obtain 50 % *para*-enriched hydrogen. A container was pressurized with 10 bar of *para*-enriched $H_{2}$ to contain gas for a whole series of experiments at 4 bar of parahydrogen pressure.

Hydrogenation experiments of disodium acetylenedicarboxylate and catalyst $[{\mathrm{Cp}}^{*}\mathrm{Ru}({\mathrm{CH}}_{3}\mathrm{CN}{)}_{3}]{\mathrm{PF}}_{6}$ were carried out strictly according to the experimental procedure in Table [Table Ch1.T1]. For each experiment, a 300 µ L aliquot was used from a stock solution. Bubbling was performed in a 5 mm Wilmad^®^ quick pressure valve NMR tube through a $1/16^{\prime \prime }$ PEEK capillary, using 4 bar parahydrogen pressure, 60 ${}^{\circ }$ C (333 K) temperature, and a gas flow of 400 sccm.

Spin locking was performed by irradiating a continuous wave rf field at the mean resonance frequency of the ${\mathrm{CH}}_{2}$ protons and with an amplitude corresponding to a 1.0 kHz nutation frequency.

## 6 Conclusions

In this work we have demonstrated *geminal* hydrogenation of a precursor molecule using *para*-enriched hydrogen gas. We show that singlet order for the methylene proton pair may be maintained by application of a spin-locking field and that the proton singlet order in the product molecule relaxes with the time constant $T_{S}^{\text{I}}≃151$ s, which is more than 50 times $T_{1}$ . We have developed a simplified kinetic model to describe the time dependence of the hyperpolarized signals observed in such experiments, which include the chemical kinetics as well as the spin dynamics. This allows simultaneous fitting of the data from several experiments and estimation of most of the kinetic parameters and relaxation rate constants.

The particular hydrogenation reaction discussed here does not lead to a product molecule with a biological function. Nevertheless, our results demonstrate the principle of methylene hyperpolarization by hydrogenative PHIP and that the short $T_{1}$ values of these protons do not necessarily prevent the accumulation of hyperpolarization. We hope that this work might allow exploration of a new range of hyperpolarized molecular targets.

## Supplement

10.5194/mr-1-175-2020-supplement The supplement related to this article is available online at: https://doi.org/10.5194/mr-1-175-2020-supplement.

## Data Availability

All 1H NMR spectra, the general pulse sequence used, as well as data points seen in the kinetics are openly available from the University of Southampton repository at https://doi.org/10.5258/SOTON/D1494 (Dagys et al., 2020).

## Appendix

The supplement related to this article is available online at: https://doi.org/10.5194/mr-1-175-2020-supplement.
