# Supplementary material for: Geminal parahydrogen-induced polarization: accumulating long-lived singlet order on methylene proton pairs
Source: Magn Reson (Gott). 2020 Aug 7;1(2):175–86. doi: 10.5194/mr-1-175-2020 (PMC10500696; doi:10.5194/mr-1-175-2020)
Supplement: The supplement related to this article is available online at: https://doi.org/10.5194/mr-1-175-2020-supplement. [file mr-1-175-supplement.pdf]

Supplement of Magn. Reson., 1, 175–186, 2020
https://doi.org/10.5194/mr-1-175-2020-supplement
© Author(s) 2020. This work is distributed under
the Creative Commons Attribution 4.0 License.

Open Access
MAGNETIC RESONANCE

*Supplement of*

# ***Geminal*** **parahydrogen-induced polarization: accumulating long-lived singlet order on methylene proton pairs**

**Laurynas Dagys et al.**

*Correspondence to:* Malcolm H. Levitt (mhl@soton.ac.uk)

The copyright of individual parts of the supplement might differ from the CC BY 4.0 License.

## S1 Chemical structure determination of I

The compound sodium 4-(carboxylatomethyl)-5-oxo-2,5-dihydrofuran- 2,3-dicarboxylate (**I**) is created in low concentration in water, on the order of few mmol during the hydrogenation of acetylene dicarboxylic acid disodium salt to form fumaric acid disodium salt using the catalyst $[Cp^{*}Ru(CH_3CN)_3]PF_6$. The low concentration of **I** made its characterization challenging. It turned out to be impossible to isolate a sufficient amount of **I** to allow a full characterization. The chemical structure of **I** was therefore confirmed by comparing its NMR characteristics with the salt **S-3** synthesized by the route shown in figure S1. As discussed below, the $^{1}$H NMR spectra of the hydrogenation product **I** and the salt c are exactly superimposed at all pH values, with a mixture of the two never displaying any additional peaks. We conclude that **I** and **S-3** are identical.

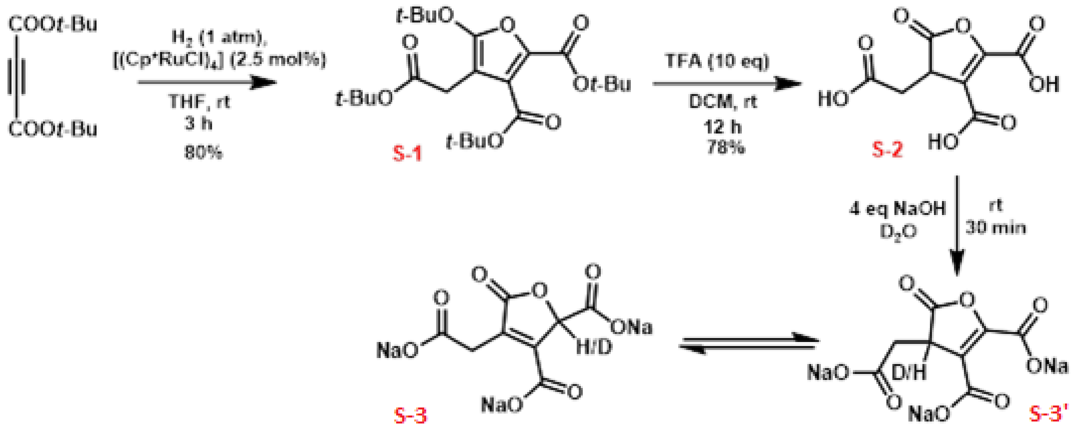


**Figure S1.** Synthetic route to the salt **S-3**, which is shown to be identical to the hydrogenation product **I**.

### S1.1 Synthesis of S-3

*Di-tert-butyl 5-(tert-butoxy)-4-(2-(tert-butoxy)-2-oxoethyl)furan-2,3-dicarboxylate (**S-1**)*
Following a reported procedure Guthertz et al. (2018), hydrogen gas was bubbled through a stirred solution of $[Cp^{*}RuCl_4]$ (48 mg, 0.0442 mmol, 2.5 mol%) in THF (15 mL) for 5 min. Di-*tert*-butyl acetylenedicarboxylate (400 mg, 1.77 mmol) was added and stirring was continued for 3 h under a $H_2$ atmosphere. For work up, all volatile materials were removed under vacuum and the residue was purified by flash chromatography (hexanes/EtOAc, 4/1) to yield compound **S-1** as pale yellow oil (321 mg, 80%). $^{1}$H NMR (500 MHz, $CDCl_3$): $\delta$ 3.32 (s, 2H), 1.54 (s, 9H), 1.53 (s, 2H), 1.41 (s, 9H), 1.40 (s, 9H). $^{13}$C NMR (126 MHz, $CDCl_3$): $\delta$ 169.7, 162.0, 157.1, 155.6, 135.2, 126.4, 100.8, 85.6, 81.9, 81.0, 29.7, 28.6, 28.2, 28.1, 28.0. ESI-MS calcd for $C_24H_39O_8$ $(M+H)^{+}$ 455.2639; found: 455.2651.

*4-(Carboxymethyl)-5-oxo-4,5-dihydrofuran-2,3-dicarboxylic acid (**S-2**)*
To a stirred solution of ester **S-1** (200 mg, 0.44 mmol) in dichloromethane (8 mL) was added TFA (337 $\mu$L, 4.4 mmol) at 0 °C. The mixture was warmed to rt and stirred for 12 h at the same temperature. The volatile materials were removed under vacuum and the residue was triturated with $Et_2O$ to afford acid **S-2** (79 mg, 78%) as a sufficiently pure colorless solid. $^{1}$H NMR (400 MHz, $(CD_3)_2CO$): $\delta$ 5.74 (dd, 1H, $J$ = 1.4, 0.8 Hz), 3.83 (dd, 1H, $J$ = 16.9, 1.4 Hz), 3.72 (dd, 1H, $J$ = 16.9, 0.8 Hz), $^{13}$C NMR (126 MHz, $(CD_3)_2CO$): $\delta$ 172.2, 169.7, 169.6, 160.9, 148.3, 133.9, 79.9, 28.0. ESI-MS calcd for $C_24H_39O_8$ $(M-H)^{-}$ 228.9990; found: 228.9971. The acid **S-2** was then converted to the corresponding tetrasodium salt **S-3** by adding 4 eq. of NaOH and stirring for 30 min in $D_2O$.

## S1.2 Structure determination of S-3

Figures S2 – S6 show the NMR spectra acquired for the determination of the chemical structure of the acid **S-2**. The molecular structure of the synthesised acid **S-2**, was determined to be 4-(carboxylatomethyl)-5-oxo-2,5-dihydrofuran- 2,3-dicarboxylate. Therefore its sodium salt **S-3** is sodium 4-(carboxylatomethyl)-5-oxo-2,5-dihydrofuran- 2,3-dicarboxylate (see figure S1).

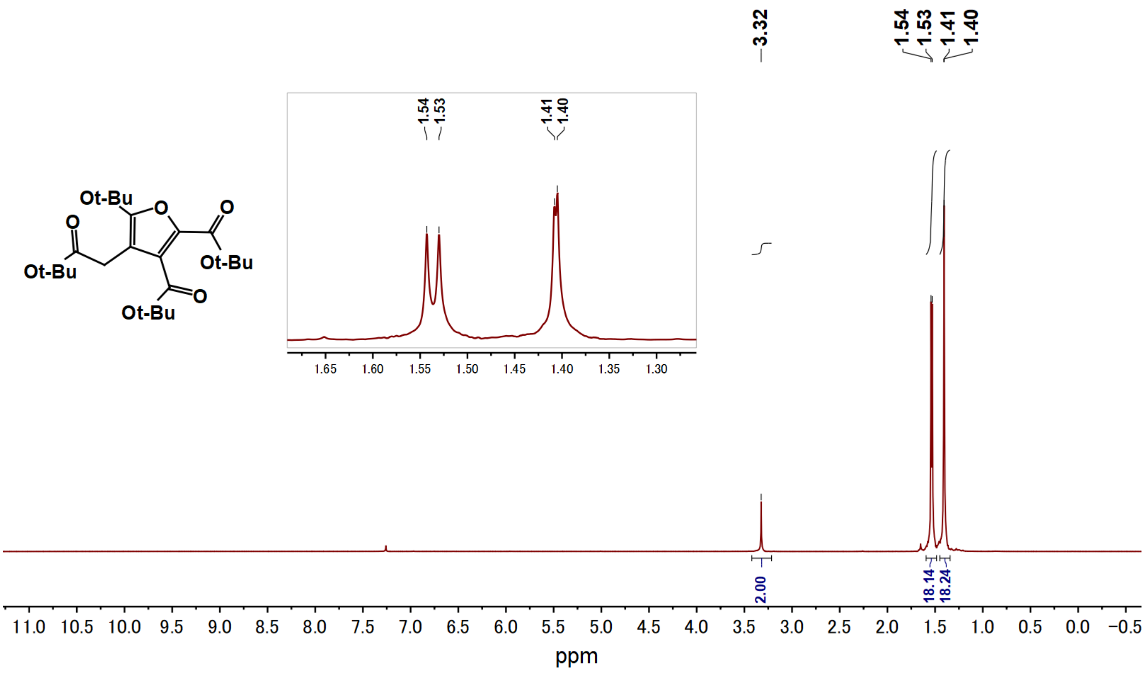


**Figure S2.** $^{1}$H-NMR spectrum of ester **S-1** (500 MHz, $CDCl_3$)

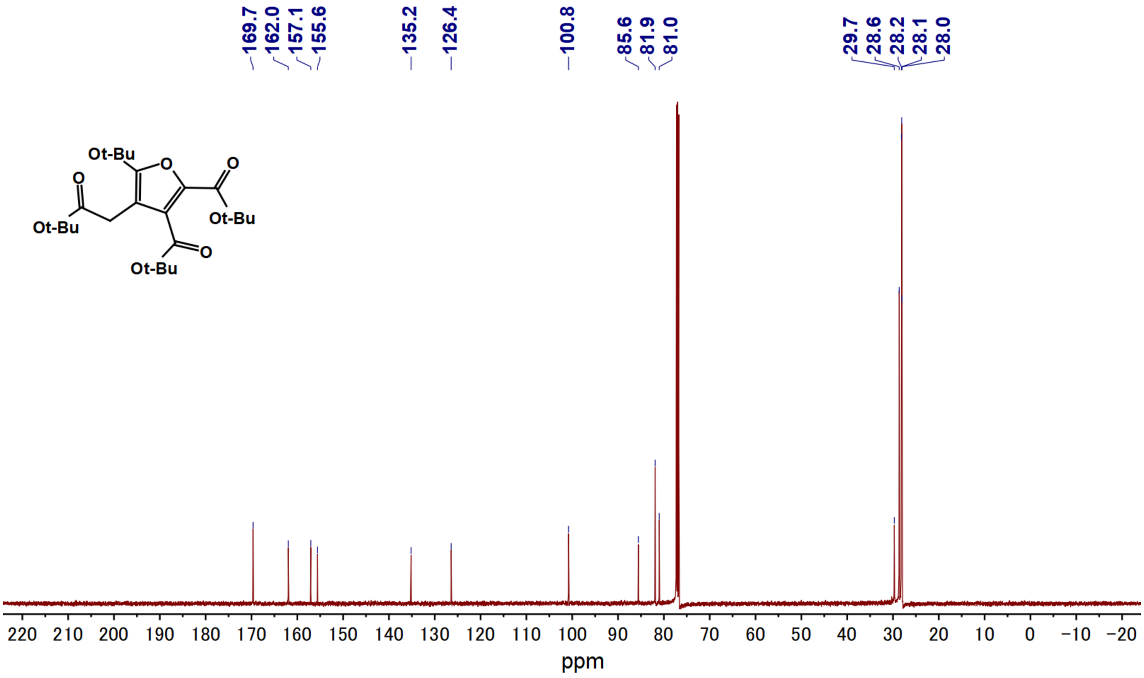


**Figure S3.** $^{13}$C-NMR spectrum of ester **S-1** (126 MHz, $CDCl_3$)

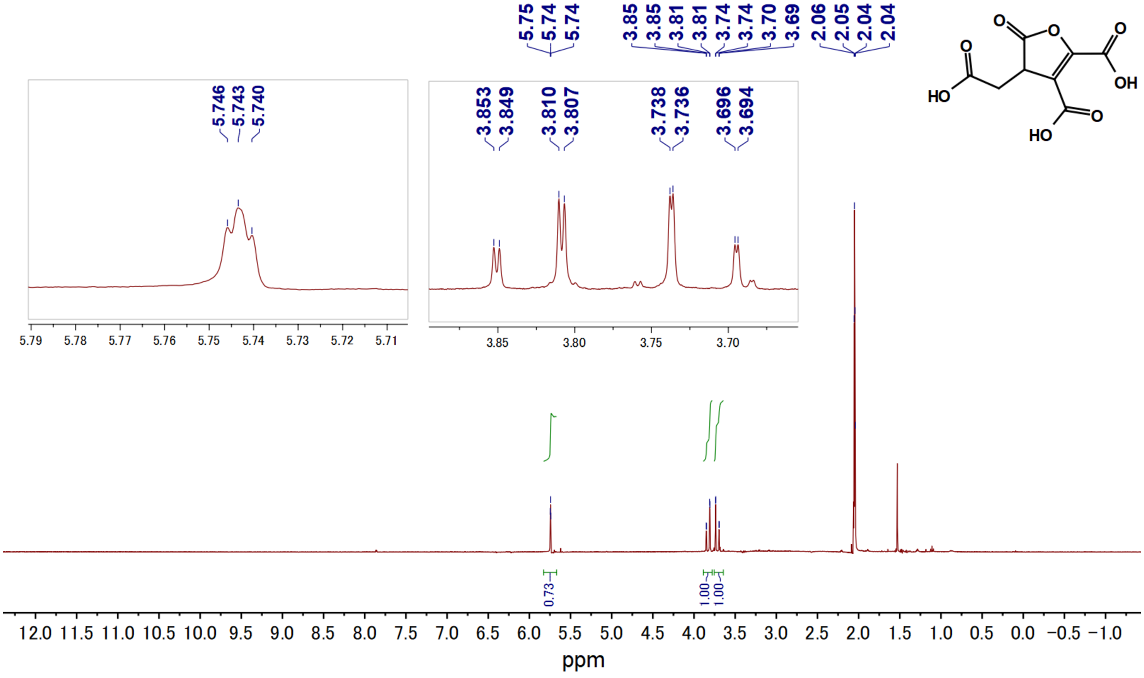


**Figure S4.** $^{1}$H-NMR spectrum of acid **S-2** (400 MHz, $(CD_3)_2CO$))

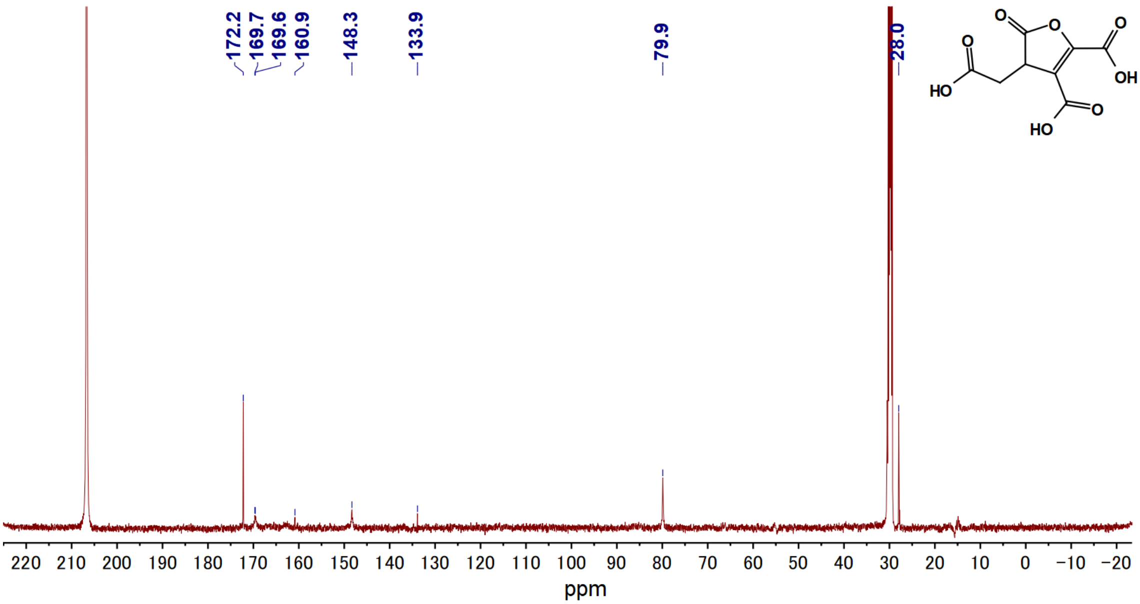


**Figure S5.** $^{13}$C-NMR spectrum of acid **S-2** (126 MHz, $(CD_3)_2CO$))

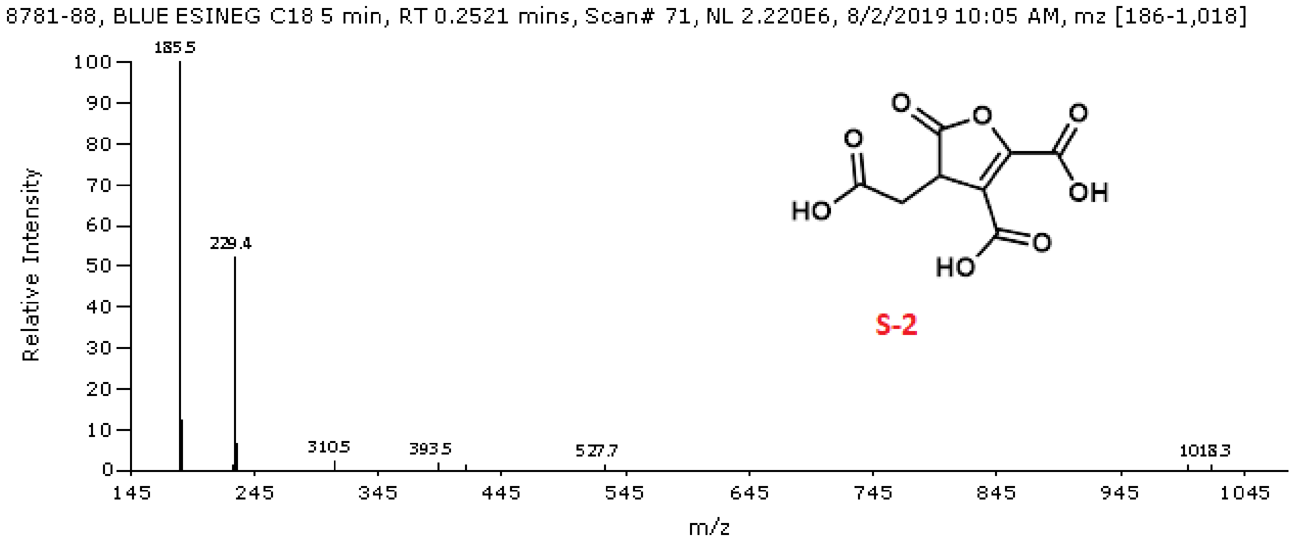


**Figure S6.** Mass spectrometry of acid **S-2**

## S1.3 Identity of S-3 and I

Figure S7 shows proton NMR spectra of the pure synthesised compound **S-3** and of a mixture of synthesised **S-3** with the reaction product solution containing the molecule **I**. Since the NMR characteristics of **S-3** and **I** are indistinguishable, it was concluded that the molecules **I** and **S-3** are identical.

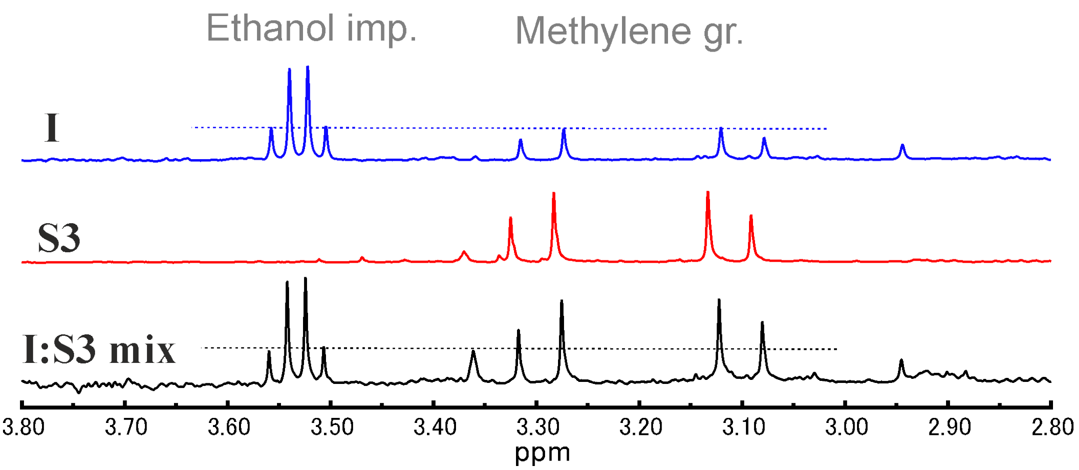


**Figure S7.** $^1$H NMR spectra of the gemPHIP product **I** (blue), the synthesised salt **S-3** (red) and the **I**:**S-3** mixture (black), all dissolved into $D_2O/H_2O$ solution. Guiding lines are given with respect to the ethanol impurity observed in the first and the last spectra. The addition of synthesised molecule only increases the signal of the methylene group protons which supports that **I** has the same identity as **S-3**.

## S2 Singlet decay time constant of the geminal protons

Samples for $T_S$ measurements with thermal polarization were prepared as follows: A hydrogenation reaction was performed by bubbling hydrogen at 6 bar pressure for 5 minutes through the sample solution at an elevated temperature of 60°C to obtain a detectable amount of **I**. Prepared samples were degassed by 5 min nitrogen bubbling at atmospheric pressure and vacuum-sealed to reduce the amount of paramagnetic oxygen. The reference sample contained 30 mM of synthesized substance **I** in $D_2O$ and was degassed identically. For experiments on thermal equilibrium samples, a recycle delay of 10 s was used between NMR experiments. The experiments were performed at a temperature of 22°C.

The time constant for singlet order decay was estimated by conventional NMR on a degassed sample at room temperature (22°C). To measure $T_S$ the singlet spin order was populated using the Sarkar pulse sequence (Sarkar et al. (2007); Levitt (2012)), followed by the application of a spin-locking field was for a variable duration. Afterwards, the remaining singlet spin-order was converted back into observable magnetization and the NMR signal acquired (see figure S8). A sufficiently strong proton decoupling field is necessary to suppress the magnetic inequivalence of the two protons (Pileio and Levitt (2009)).

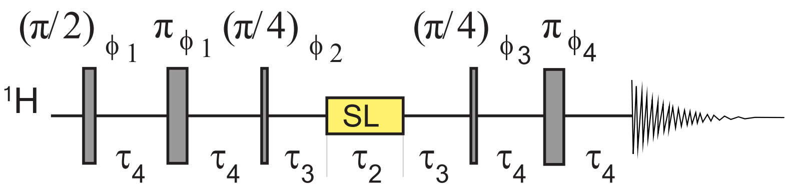


**Figure S8.** Pulse sequence used for determination of singlet state lifetime $T_S$ on thermally polarized samples. The singlet spin order was populated using the Sarkar pulse sequence, then a spin-locking field was applied for a variable duration, followed by reconversion of the singlet spin order into observable magnetization and acquisition of the NMR signal. A phase cycle given by $\phi_1 = $ [x, y, -x, -y], $\phi_2 = $ [y, -x, -y, x], $\phi_3 = $ [y, y, y, y] and $\phi_4 = $ [x, x, x, x] is used to filter out unwanted signal components created by the initial Sarkar sequence. The timings are given by: $\tau_4 = 1/4J$ and $\tau_3 = \pi/\omega_\Delta$, where $\omega_\Delta$ is chemical shift different between two nuclear spins.

We have determined $T_S$ = 61.1 $\pm$ 7.1 s and $T_1$= 1.23 $\pm$ 0.14 s at 9.41 T for a reaction product solution containing in average between 60 and 70 mM precursor disodium acetylenedicarboxylate, between 20 and 30 mM reaction product disodium fumarate, up to 10 mM catalytic side product **I** and 6 mM catalyst $[Cp^*Ru(CH_3CN)_3]PF_6$ in $D_2O$. Results are shown in figure S9. For comparison, the measurements were repeated on a degassed solution containing only 30 mM of the chemically synthesized version of **I** in $D_2O$. The results were found to agree within experimental error. We have, thus, negated potentially problematic factors, such as relaxation due to the presence of a catalyst.

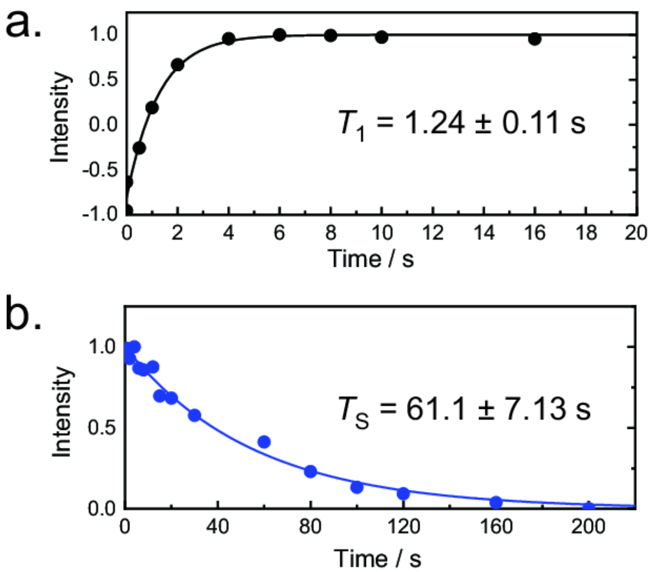


**Figure S9.** Determination of $T_1$ (a.) and $T_S$ (b.) using inversion recovery and pulse sequence from figure S8, respectively. Experiments were carried-out in 9.41 T magnetic field at 22°C. The estimated values are given in the plots. Note the difference between the timescales of the experiments.

In order to determine which spin-locking amplitude is sufficient to reach the near equivalence regime, where the influence of the chemical shift difference between the protons is suppressed, the singlet decay experiment was carried out with different

spin-locking powers and at different magnetic fields. The singlet state relaxation rate $R_S$ $(= 1/T_S)$ is expected to converge asymptotically towards a minimum value where the decoupling is strong enough to be used for singlet state lifetime determination. Figure S10 shows the convergence of $T_S^{-1}$ to a minimum value for large spin-locking nutation frequencies, corresponding to maximum $T_S$ times of 39 s at 16.4 T and 61 s at 9.41 T. The results indicate that a spin-locking nutation frequency of 1 kHz is sufficient to reach the near-equivalence regime at 9.41 T. The $T_1$of the protons was measured by inversion recovery experiments on the same sample at 9.41 T, and found to be $T_1$= 1.23 ± 0.14 s, a factor of 50 shorter than $T_S$.

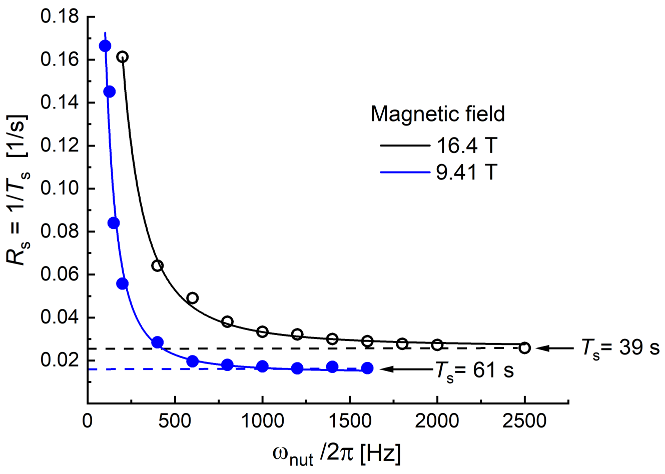


**Figure S10.** Singlet order relaxation rate constant $R_S = 1/T_S$ plotted against spin-locking nutation frequencies $\nu_{nut} = \omega_{nut}/2\pi$ in Hz. The value of $T_S^{-1}$ converges to a field-dependent minimum value, corresponding to maximum $T_S$ times of 39 s at 16.4 T and 61 s at 9.41 T. The near equivalence regime is reached for spin-locking nutation frequencies from 1 kHz upwards. The measurements were carried out on thermally polarized and degassed samples at room temperature (22°C).

## S3 Decomposition of product I

At sufficiently high temperature, molecule **I** decomposes into a simpler form with one carboxylic group missing, as depicted in figure S11. The decarboxylation process takes around 2 hours at 333 K but is accelerated at higher temperatures. Since the chemical structure of **II** is achiral, its methylene protons are chemically equivalent, in contrast to those of the chiral compound **I**. A $^1$H NMR spectrum showing peaks from the methylene protons of both species is shown in the appendix of the manuscript.

**I** **II**

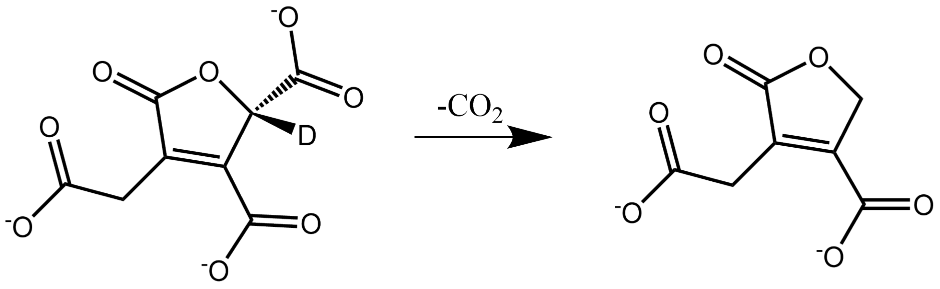


**Figure S11.** Decarboxylation of the chiral product molecule **I** into the achiral compound **II**.

## References

Guthertz, A., Leutzsch, M., Wolf, L. M., Gupta, P., Rummelt, S. M., Goddard, R., Farès, C., Thiel, W., and Fürstner, A.: Half-Sandwich Ruthenium Carbene Complexes Link Trans-Hydrogenation and Gem-Hydrogenation of Internal Alkynes, J. Am. Chem. Soc., 140, 3156–3169, 2018.

Levitt, M. H.: Singlet Nuclear Magnetic Resonance, Annu. Rev. Phys. Chem., 63, 89–105, 2012.

Pileio, G. and Levitt, M. H.: Theory of Long-Lived Nuclear Spin States in Solution Nuclear Magnetic Resonance. II. Singlet Spin Locking, J. Chem. Phys., 130, 214 501–14, 2009.

Sarkar, R., Vasos, P. R., and Bodenhausen, G.: Singlet-State Exchange NMR Spectroscopy for the Study of Very Slow Dynamic Processes, J. Am. Chem. Soc., 129, 328–334, 2007.
